# A reliable and reproducible method for establishing accurate patella drill holes for quadriceps tendon repair

**DOI:** 10.1308/003588413X13511609957056e

**Published:** 2013-01

**Authors:** A Dinneen, J Clements, G Heilpern, G Railton

**Affiliations:** Kingston Hospital NHS Trust, UK

## Background

Various techniques for quadriceps tendon repair have been described with biomechanical studies suggesting greater failure threshold with longitudinally placed drill holes.^1^ The current gold standard for repair uses 3–4 sutures^2^ passed through parallel longitudinal transpatellar bone tunnels. We describe a simple and reproducible technique for accurate placement of patella drill holes and passage of suture material.

## Technique

**Figure 1:**
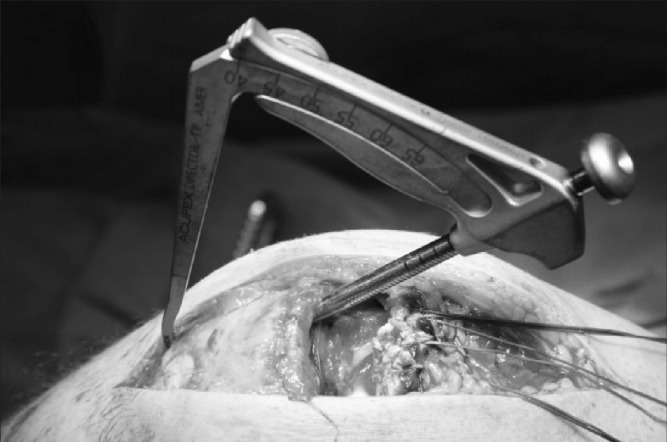
Acufex^®^ Director™ Drill Guide positioned on patella

**Figure 2:**
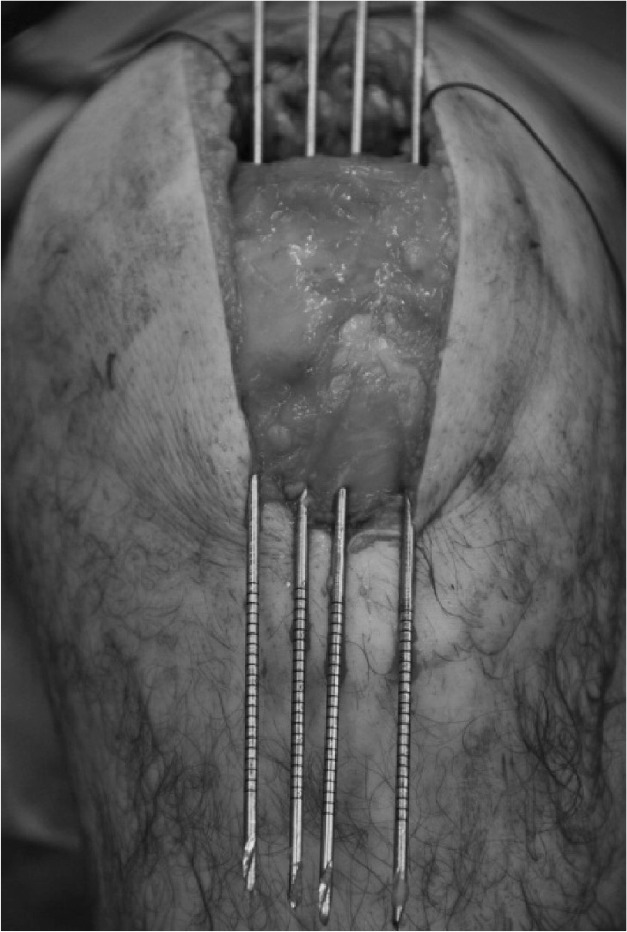
2.4mm drill tip passing pins through patella

**Figure 3:**
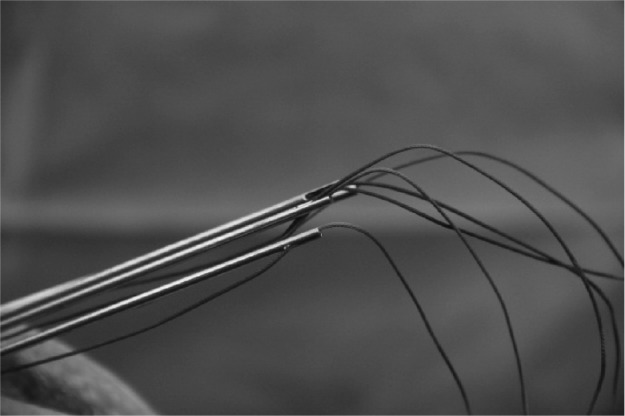
Sutures passed through eyes of 2.4mm drill tip passing pins

**Figure 4:**
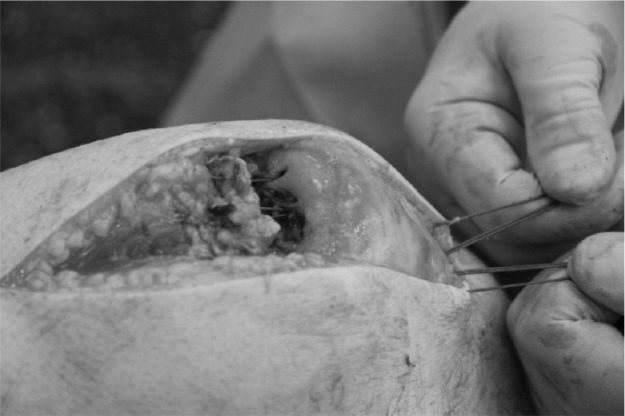
Sutures pulled through patella tunnels to inferior pole

Satisfactory hold is achieved in the quadriceps tendon with sutures. Using the Acufex^®^ Director™ Drill Guide (Smith & Nephew, Andover, MA, US), the desired entry and exit locations for drill holes are selected on the patella (Fig 1). Three to four 2.4mm drill tip passing pins are placed (Fig 2). Each passing pin is then overdrilled with the 4.5mm ENDOBUTTON^®^ (Smith & Nephew) cannulated drill bit. The free ends of suture are passed through the eyes of the 2.4mm drill tip passing pins (Fig 3) and pulled through the tunnels, exiting at the inferior pole (Fig 4). The repair can be tensioned as required before tying the suture ends at the inferior pole of the patella.

## Discussion

The ability to drill parallel longitudinal transpatellar bone tunnels accurately freehand avoiding the articular surface while maintaining accurate exit points can pose a technical challenge. Coupled with difficulties encountered in passing the sutures through the tunnels, this can result in prolonged operative time with an associated increase in morbidity and needless frustration for the operating surgeon. The technique described here overcomes these difficulties while achieving gold standard repair.
